# Incommensurate magnetic structure of CrAs at low temperatures and high pressures

**DOI:** 10.1107/S205252062300817X

**Published:** 2023-10-12

**Authors:** Andreas Eich, Andrzej Grzechnik, Yixi Su, Bachir Ouladdiaf, Denis Sheptyakov, Thomas Wolf, Vaclav Petricek, Hend Shahed, Karen Friese

**Affiliations:** a Jülich Centre for Neutron Science-2 and Peter Grünberg Institute-4 (JCNS-2/PGI-4), Forschungszentrum Jülich, 52425, Germany; b Institute of Crystallography, RWTH Aachen University, Aachen, 52066, Germany; c Jülich Centre for Neutron Science-4 (JCNS-4), Forschungszentrum Jülich, 52425, Germany; d Jülich Centre for Neutron Science (JCNS), Heinz Maier-Leibnitz Zentrum, Garching, 85747, Germany; e Institut Laue–Langevin, Grenoble, 38000, France; f Laboratory for Neutron Scattering and Imaging, Paul Scherrer Institute, Villigen, 5232, Switzerland; g Institute for Quantum Materials and Technologies, Karlsruhe Institute of Technology, Karlsruhe, 76021, Germany; h Institute of Physics of the Czech Academy of Sciences, Prague 8, 182 00, Czech Republic; Adam Mickiewicz University, Poland

**Keywords:** incommensurate magnetic structure, neutron diffraction, extreme conditions, high pressure

## Abstract

The magnetic structure of chromium arsenide CrAs is studied with neutron powder diffraction at ambient pressure in the temperature range 1.5–300 K as well as with neutron single-crystal diffraction at 2 K and 0.12 GPa. The material undergoes an anti-isostructural phase transition at *T*_N_ = 267 K and atmospheric conditions, in which both orthorhombic phases have the same space-group symmetry (*Pnma*, *Z* = 4) but different distortions of the parent hexagonal structure of the NiAs type (*P*6_3_/*mmc*, *Z* = 2). The magnetic structure below *T*_N_ is incommensurate with the propagation vector **k** = (0, 0, *k*_c_). At ambient pressure, the component *k*_c_ decreases from *k*_c_ = 0.3807 (7) at 260 K to *k*_c_ = 0.3531 (6) at 50 K. Below this temperature, it is basically constant. With increasing pressure at 2 K, *k*_c_ is also constant within standard uncertainties [*k*_c_ = 0.353 (2)]. For the analysis of the magnetic structure, a group-theoretical approach based on the space group of the nuclear structure and its subgroups is used. To avoid falling into false minima in the refinements, a random search for magnetic moments in the models is implemented. In the literature, the magnetic structure has been determined on the basis of powder diffraction data as a double helix propagating along the *c* axis. Although this double-helical model leads to satisfactory agreement factors for our powder data, it does not reproduce the intensities of the magnetic satellite reflections measured on single-crystal data in a satisfactory way and can therefore be discarded. Instead, several other models are found that lead to better agreement. Each of them is spiral-like with directional components in all three directions and with no spin-density wave character that would cause a non-constant magnetic moment. In all these models, the ordering of the spins is neither a pure helix nor a pure cycloid. Instead, the unit vectors of the spin rotation planes make an angle α, 0° < α < 90°, with respect to the c* direction. The model in superspace group *P*2_1_.1′(α0γ)0*s* yields the best agreement factors in the refinements of the neutron single-crystal and powder diffraction data. This model is unique as it is the only one in which all the magnetic moments rotate with the same chirality.

## Abstract 2

The incommensurate magnetic structure of chromium arsenide CrAs below the anti-isostructural phase transition is studied with neutron powder and single-crystal diffraction at high pressures and low temperatures.

## 1. Introduction

Chromium arsenide CrAs is considered a model system to study the interplay of unconventional superconductivity and the helimagnetic order (Cheng & Luo, 2017 ▸). At ambient conditions, it crystallizes in the MnP-type structure (*Pnma*, *Z* = 4) which is a distorted variant of the NiAs-type structure (*P*6_3_/*mmc*, *Z* = 2) (Rundqvist *et al.*, 1962 ▸; Tremel *et al.*, 1986 ▸).

At 267 K, CrAs undergoes a first-order phase transition from the room-temperature paramagnetic phase to a low-temperature antiferromagnetically ordered phase that is incommensurate (Watanabe *et al.*, 1969 ▸). On lowering the temperature, the incommensurate propagation vector decreases from **k** = 0.38c* at 265 K to **k** = 0.36c* at 1.5 K, which has been linked to a weakening of the Dzyaloshinskii–Moriya interactions between Cr atoms (Pan *et al.*, 2020 ▸). The magnetic transition at *T*_N_ ≃ 267 K is accompanied by abrupt changes in the unit-cell parameters Δ*a*/*a* ≃ −0.4%, Δ*b*/*b* ≃ +3.5% and Δ*c*/*c* ≃ −0.8% as well as in the unit-cell volume Δ*V*/*V* ≃ +2.25%. On the basis of single-crystal diffraction data, it has been demonstrated that CrAs below and above the phase transition has the same space-group symmetry *Pnma*, *Z* = 4 (Eich *et al.*, 2021 ▸). At *T*_N_ ≃ 267 K, the *c*/*b* axial ratio, which is close to the ideal value of 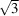 related to the orthohexagonal setting of the parent hexagonal structure, abruptly changes from *c*/*b* > 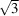 to *c*/*b* < 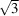 . Compressing CrAs across *T*_N_ at low temperatures is equivalent to warming up the material from the magnetically ordered to paramagnetic phases at atmospheric pressure (Eich *et al.*, 2021 ▸; Grzechnik *et al.*, 2023 ▸). The structural phase transition at *T*_N_ is of the anti-isostructural type, in which both orthorhombic phases have the same space-group symmetry (*Pnma*, *Z* = 4) but different distortions of the parent hexagonal structure of the NiAs type (*P*6_3_/*mmc*, *Z* = 2). Associated with such a phase transition is the development of a twinned microstructure. The pressure dependence of *T*_N_ inferred from synchrotron single-crystal data (Grzechnik *et al.*, 2023 ▸) agrees with the phase diagram drawn by Shen *et al.* (2016 ▸).

On compression, the magnetic transition is completely suppressed above a critical pressure of *p*_c_ ≃ 0.7 GPa (Kotegawa *et al.*, 2014 ▸; Matsuda *et al.*, 2018 ▸) due to the stabilization of the lower-volume paramagnetic phase with pressure. At all pressures, the magnetic and the structural transitions are coupled (Matsuda *et al.*, 2018 ▸) and the observed hysteresis indicates that the structural transition remains of first order up to its suppression.

Above about 0.3 GPa (Kotegawa *et al.*, 2014 ▸; Wu *et al.*, 2014 ▸; Shen *et al.*, 2016 ▸), CrAs exhibits a dome-like-shaped superconducting phase region with a maximum *T*_c_ ≃ 2.2 K at about 1.0 GPa. At higher pressures, the critical temperature decreases again, until the superconducting phase is suppressed at about 4.4 GPa (Matsuda *et al.*, 2018 ▸). Between the onset of superconductivity at 0.3 GPa and the suppression of magnetic order at 0.7 GPa, a two-phase region with competing magnetic and superconducting properties is observed (Keller *et al.*, 2015 ▸; Khasanov *et al.*, 2015 ▸). The nature of the superconductivity in CrAs is not yet fully understood (Cheng & Luo, 2017 ▸), with different results supporting either conventional (Khasanov *et al.*, 2015 ▸) or unconventional (Wu *et al.*, 2014 ▸; Kotegawa *et al.*, 2015 ▸; Shen *et al.*, 2016 ▸; Nigro *et al.*, 2019 ▸) pairing mechanisms. The authors claiming unconventional superconductivity assume that the pairing is mediated by antiferromagnetic fluctuations between nearest-neighbour Cr atoms (Chen & Wang, 2019 ▸; Shen *et al.*, 2016 ▸).

Up to now, most studies on CrAs in and near the superconducting phase region have been focused on its magnetic (Wu *et al.*, 2014 ▸; Khasanov *et al.*, 2015 ▸; Kotegawa *et al.*, 2015 ▸; Matsuda *et al.*, 2018 ▸) and transport (Kotegawa *et al.*, 2014 ▸; Wu *et al.*, 2014 ▸; Kim *et al.*, 2017 ▸) properties. The magnetic structure of CrAs, however, has not been thoroughly investigated as a function of both temperature and pressure. While the observed temperature dependence of the propagation vector **k** is reported rather consistently in various studies (Selte *et al.*, 1971 ▸; Matsuda *et al.*, 2018 ▸; Keller *et al.*, 2015 ▸; Shen *et al.*, 2016 ▸), the influence of pressure on the propagation vector is debatable. At effectively the same temperatures (1.5–4 K), different studies show either a decrease [the data measured on a single crystal by Matsuda *et al.* (2018 ▸) and the data measured on a powder by Shen *et al.* (2016 ▸)] or an increase of the *k*_c_ component of the propagation vector [data measured on a powder by Keller *et al.*, 2015 ▸)] with increasing pressure. According to Keller *et al.* (2015 ▸), the change of the **k** vector is accompanied by a spin reorientation. The discrepancies concerning the spin reorientation (Matsuda *et al.*, 2018 ▸) have been related to the form of the sample (*i.e.* single-crystalline versus polycrystalline) but a precise explanation for this was not elaborated. However, as differences are even observed when comparing two powder measurements, it is obvious that this explanation cannot be sufficient to explain the differences. The ambiguity with respect to the behaviour of the **k** vector implies that the influence of pressure on the magnetic structure of CrAs is far from being understood.

It should be noted that the magnetic structure of CrAs has up to now not been determined in a rigorous way. Originally, it was proposed that, as the helical model for the magnetic structure of MnP allowed the indexing of the satellite reflections in CrAs, the magnetic structure of CrAs was basically identical to the one observed in MnP (Watanabe *et al.*, 1969 ▸). Accordingly, it was supposed that four helices are formed, which can be separated into two in-phase pairs with a fixed angle between them. Consequently, the structure was described as double helical (Chen & Wang, 2019 ▸). However, it is well known from other systems that isostructural compounds with different paramagnetic ions exhibit different magnetic interactions and therefore do not necessarily lead to comparable arrangements of spins in the ordered structures. Since the emergent magnetic structure depends directly on the specific magnetic properties of the magnetic atoms and their interactions, and indirectly also on the underlying electronic configurations, the similarity of the magnetic structures of CrAs and MnP cannot be assumed *a priori*. While a similar magnetic structure is certainly plausible, alternative magnetic structures, which are in accordance with symmetry considerations, cannot be dismissed outright.

The model proposed by Watanabe *et al.* (1969 ▸) was afterwards followed by other authors (Selte *et al.*, 1971 ▸; Keller *et al.*, 2015 ▸), who refined the magnetic structure on the basis of powder data. In none of the refinements of the magnetic structure was the concept of magnetic superspace groups employed. However, in an entry for the magnetic structure of CrAs in the database MAGNDATA (Gallego *et al.*, 2016 ▸), the magnetic superspace group *P*2_1_2_1_2_1_.1′(00γ)00*ss* is given as the one corresponding to the reported structure by Watanabe *et al.* (1969 ▸) and Selte *et al.* (1971 ▸). The double-helix model includes restrictions that are not forced by the symmetry of *P*2_1_2_1_2_1_.1′(00γ)00*ss*.

To our knowledge, a determination of the magnetic structure of CrAs based on group-theoretical considerations, including a comparison of all symmetry-allowed models for its magnetic structure, was performed neither by Watanabe *et al.* (1969 ▸) nor in any of the subsequent publications on this subject. In addition, investigations based on complete single-crystal data have not been performed up to now. The purpose of our work was to reinvestigate the magnetic structure of this material with neutron powder and single-crystal diffraction at high pressures and low temperatures in the vicinity of the superconducting phase and to determine its superspace symmetry. For the analysis of the magnetic structure, we adopted a group-theoretical approach based on the space group of the nuclear structure (and its subgroups) and the propagation vector. Our approach is based on the concept of superspace symmetry that rationalizes incommensurate magnetic structures (Perez-Mato *et al.*, 2012 ▸) and on the classification of the magnetic superspace groups compatible with the helical and/or cyclo­idal magnetic modulations (Fabrykiewicz *et al.*, 2021 ▸). The analysis and refinements of both polycrystalline and single-crystalline data were carried out using the program *Jana2020* (Petříček *et al.*, 2023 ▸).

## 2. Experimental

The sample preparation is described by Eich *et al.* (2021 ▸).

Neutron single-crystal diffraction at low temperatures and high pressures was performed using a TiZr clamp cell (03PCL150TZ5) on the beamline D9 at the Institut Laue–Langevin (ILL, Grenoble, France). The sample was an as-grown CrAs single crystal that was cut to the length of 3 mm along its growth *a* axis and had a final size of 3 × 2 × 2 mm. The quality and orientation of the sample were checked using a neutron Laue camera (OrientExpress at ILL). The crystal was glued onto a small cylinder made from aluminium to preserve the orientation when inserted into the sample capsule. The *a* axis was parallel to the clamp-cell axis (vertical when in the cryostat) and the (*b*, *c*) plane was perpendicular to it. The sample capsule was made of aluminium and filled with Fluorinert FC770 as a pressure-transmitting medium. Pressure was applied using a stationary wheel-driven hydraulic press. The initial pressure at room temperature was determined to be 0.17 GPa. The loaded cell was inserted into an orange-type cryostat and cooled to 2 K. Based on earlier experiences, the pressure drop upon cooling amounts to approximately 30% for this temperature decrease (Lelièvre-Berna, 2023 ▸). Accordingly, the first data collection was performed at 0.12 GPa and 2 K. The measurement consisted of a series of ω scans. Data collection out of the horizontal reflection plane was possible due to the lifting counter range of the detector, −12.5° to 25°. Afterwards, the cell was warmed to room temperature and compressed to 1.2 GPa. After cooling it to 2 K, this corresponds to a pressure of about 0.84 GPa. Under these conditions, the same set of nuclear and magnetic reflections, like at 0.12 GPa and 2 K, was measured with the same ω scans for comparability. The number of the observed reflections (all/main/satellites) at 0.12 GPa and 0.84 GPa (both at 2 K) were 424/378/46 and 276/229/47, respectively.

Neutron powder diffraction data (using λ = 2.45 Å and λ = 1.494 Å) were measured in the temperature range 1.5–300 K at ambient pressure on the HRPT diffractometer at the neutron spallation source SINQ (Fischer *et al.*, 2000 ▸) at the Paul Scherrer Institute (Villigen, Switzerland). About 6 g of the powder sample were placed into a sample container made of vanadium. The sample container was continuously spinning around an axis perpendicular to the scattering plane to minimize the effects of inhomogeneity and preferred orientation.

Further details of neutron single-crystal and powder data analysis are given in the supporting information.

## 3. Results

### 3.1. Neutron single-crystal diffraction at high pressures and low temperatures

Our initial goal was to determine the crystal and magnetic structures of superconducting CrAs. However, we were not able to reach the corresponding pressure and temperature conditions using the available clamp cell and cryostat. Instead, we attempted to study the magnetic structure of CrAs in the vicinity of the superconducting phase at two different pressures (0.12 GPa and 0.84 GPa) and 2 K. These conditions are in the stability field of the antiferromagnetically ordered phase observed at low temperatures and ambient pressure, for which the double-helical model is established in the literature (Kotegawa *et al.*, 2017 ▸; Shen *et al.*, 2016 ▸; Matsuda *et al.*, 2018 ▸).

Based on our neutron single-crystal diffraction data, the propagation vector of **k** = [0, 0, 0.353 (2)] indexes the satellite reflections at both pressure points and 2 K. Four models, corresponding to four different magnetic superspace groups, are deduced by the combination of the space group of the crystal structure (*Pnma*, *Z* = 4) and this propagation vector. [As in our earlier investigations on the temperature and pressure dependence of the crystal structure of CrAs, we observed the formation of three twin domains related by a threefold rotation around the *a* axis coinciding with the first-order transition at *T*_N_ (Eich *et al.*, 2021 ▸; Grzechnik *et al.*, 2023 ▸); we also used the corresponding twin model for the refinements here.] For none of these could a satisfactory agreement be reached (see Table S1 in the supporting information). We therefore lowered the symmetry of the nuclear structure to its *translationengleiche* subgroups (down to *t* = 8, see Fig. S3). This way, depending on the chosen symmetry of the subgroup, additional magnetic superspace groups were derived and tested. We also included a model corresponding to the double-helix structure reported in the literature (Watanabe *et al.*, 1969 ▸; Selte *et al.*, 1971 ▸).

In all the lower-symmetrical descriptions, the higher *Pnma* symmetry of the crystal structure was retained by fixing the respective atomic coordinates using local symmetry operations. This restriction was applied because in former structural studies based on synchrotron single-crystal data no indication of a symmetry lowering of the nuclear structure was detected (Eich *et al.*, 2021 ▸; Grzechnik *et al.*, 2023 ▸). In addition, trial refinements using our neutron single-crystal data also did not show any significant deviation of the nuclear structure from the *Pnma* symmetry. Altogether, 31 incommensurate magnetic models derived from space group *Pnma* and its subgroups were subsequently refined. It should be noted that we used the option to perform a random search for magnetic moments for the initial models (as implemented in *Jana2020*) to avoid falling into false minima in the refinement.

Details on the free magnetic parameters and the overall agreement factors for all models at 0.12 GPa and 2 K are given in the supporting information (Tables S1 and S2). An overview of the final agreement factors for the models leading to the best agreement factors is given in Table 1 ▸.

An inspection of the models derived on the basis of the non-centrosymmetric orthorhombic subgroups (*P*2_1_ *ma*, *Pn*2_1_ *a*, *Pnm*2_1_, *P*2_1_2_1_2_1_) of *Pnma* shows that the agreement factors for the satellite reflections are not satisfactory, including the magnetic superspace group *P*2_1_2_1_2_1_.1′(00γ)00*ss*) (Table S2). [This magnetic superspace group corresponds to the symmetry assigned to the double-helical model by Watanabe *et al.* (1969 ▸) and Selte *et al.* (1971 ▸). Our refinement does not include any restraints, which are not forced by the symmetry (Gallego *et al.*, 2016 ▸).] The models derived from the centrosymmetric monoclinic subgroups (*P*2_1_/*n*, *P*2_1_/*m*, *P*2_1_/*a*) show in comparison better results, with both superspace groups *P*2_1_/*n*.1′(0βγ)0*ss* and *P*2_1_/*a*.1′(00γ)*s*0*s* yielding significantly lower agreement factors for the satellite reflections (Table S2). On reduction of the symmetry to the non-centrosymmetric monoclinic subgroups (*Pa*, *Pn*, *P*2_1_^[100]^, *Pm*, *P*2_1_^[010]^, *Pn*, *P*2_1_^[001]^, where the superscripts indicate the direction of the 2_1_ axis), the agreement factors for the derived models are in general lower, and four magnetic superspace-group symmetries lead to a particularly good fit with the *wR*(obs) agreement factors for the satellites below 20%: *Pn*.1′(0βγ)*ss*, *P*2_1_.1′(0βγ)0*s* (2_1_ in direction [100]), *P*2_1_.1′(α0γ)0*s* (2_1_ in direction [010]) and *Pa*.1′(00γ)0*s* (Table 1 ▸). The triclinic subgroups 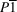 and *P*1 lead to magnetic models with similar agreement factors; however, they involve a significantly higher number of free magnetic parameters and we therefore do not consider them to be substantially better (Table 1 ▸).

One common feature of the models in *P*2_1_/*n*.1′(0βγ)0*s* *, Pn*.1′(0βγ)*ss*, *P*2_1_.1′(0βγ)0*s*, *P*2_1_.1′(α0γ)0*s* and *Pa*.1′(00γ)0*s* is the lack of any restriction in the directional components of the magnetic moment, indicating consistently that the magnetic structure cannot be described with a collinear or coplanar arrangement of the spins. This is confirmed by the fact that the higher-symmetrical magnetic models, which involve restrictions forcing the magnetic moments to lie in a specific direction or plane, lead to substantially higher agreement factors.

In addition to the refined models, which follow from symmetry considerations, the double-helix model from the literature (Watanabe *et al.*, 1969 ▸; Selte *et al.*, 1971 ▸) was considered. For this, the nuclear structure was described in space group *P*1 and additional constraints for the magnetic moments enforcing the double helix were introduced. However, our single-crystal data show conclusively that this model can definitely be discarded as the agreement factors for the satellite reflections are very high (Table 1 ▸).

In the refinements so far, the only restraints on the magnetic models were the ones posed by the symmetry of the respective magnetic superspace group. For the models in *P*2_1_/*n*.1′(0βγ)0*ss*, *Pn*.1′(0βγ)*ss*, *P*2_1_.1′(0βγ)0*s*, *P*2_1_.1′(α0γ)0*s*, *Pa*.1′(00γ)0*s*, *P* 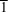 .1′(αβγ)0*s* and *P*1.1′(αβγ)0*s* further refinements with additional restraints were performed, which led to a further reduction of the magnetic parameters in the refinement (Table 1 ▸). These are: (i) the symmetrically independent Cr atoms carry an equal, but not constant, magnetic moment (‘*M*_equal_’); (ii) the absolute value of the magnetic moments of all Cr atoms is constant and the magnetic moments are only allowed to rotate (‘*M*_rot_’).

These restraints can be taken as valid assumptions as all Cr atoms in the crystal structure of CrAs are equivalent and thus indistinguishable. As expected, these restraints lead to worse overall agreement factors. In general, the restraint to a rotation of the magnetic moments has a larger effect than the equalization of the magnetic moments.

The restraints for the modulated magnetic moments of the Cr atoms allow us to narrow down the possible models to those where the maximum magnetic moment does not violate the theoretical limit of 3.87 μ_B_ for Cr^3+^ (as present in CrAs). Of the models fulfilling this condition, only those with the best agreement factors are considered in the following: *P*2_1_/*n*.1′(α0γ)0*s*, *P*2_1_.1′(0βγ)0*s*, *P*2_1_.1′(α0γ)0*s*, *Pa*.1′(00γ)0*s* and *P* 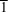 .1′(αβγ)0*s* (Table 1 ▸). [We discarded the model in *P*1.1′(αβγ)0*s* as it involves a significantly higher number of parameters, but does not lead to substantially better agreement factors.] The symmetry operations for these magnetic superspace groups are shown in Table 2 ▸. It should be noted that all of them include the operator *x*_1_, *x*_2_, *x*_3_, *x*_4_ + ½, −*m*, which ensures that all the non-modulated contributions to the magnetic ordering are fixed to zero. A comparison of all the different models shows that additional restrictions have the smallest effect on the agreement factors for the refinement in *P*2_1_.1′(α0γ)0*s*.

Refinements of the nuclear structure using the data measured at 0.84 GPa lead to substantially worse agreement factors than for the lower-pressure point due to strong broadening of the reflections with increased pressure. Since already the agreement factors for the nuclear structure are not satisfactory for this pressure point, we abstained from a refinement of the magnetic structure.

### 3.2. Neutron powder diffraction at low temperatures

**Figure 1:**
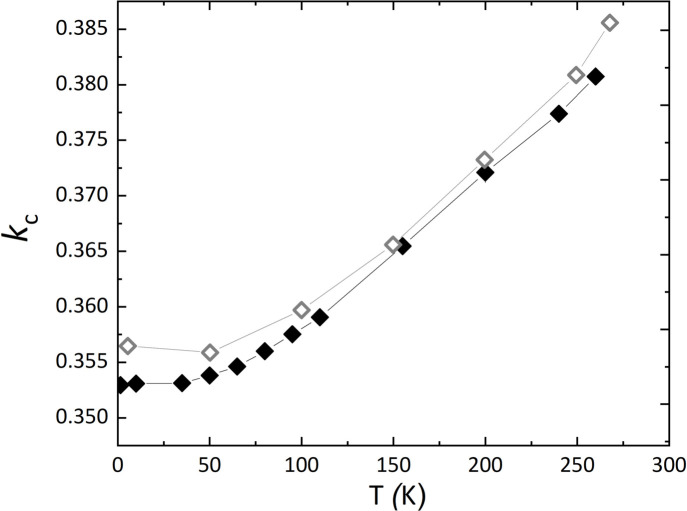
The *k*_c_ component of the propagation vector of the magnetic structure as a function of temperature determined from neutron powder diffraction experiments (full symbols) compared with the values reported by Shen *et al.* (2016 ▸) (open symbols). The standard uncertainties in our data are smaller than the size of the symbols.

Neutron powder diffraction patterns at two selected temperatures and ambient pressure are shown in Fig. S1. Traces of the high-temperature phase with *c*/*b* > 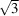 are observed even at 1.5 K. All the patterns measured below *T*_N_ = 267 K can be indexed with the propagation vector **k** = (0, 0, *k*_c_). The component *k*_c_ decreases from *k*_c_ = 0.3807 (7) at 260 K to *k*_c_ = 0.3531 (6) at 50 K; below this temperature it is basically constant (Fig. 1 ▸). Our observations are in good agreement with those made by Shen *et al.* (2016 ▸), if one considers a systematic offset between the respective values.

**Figure 2:**
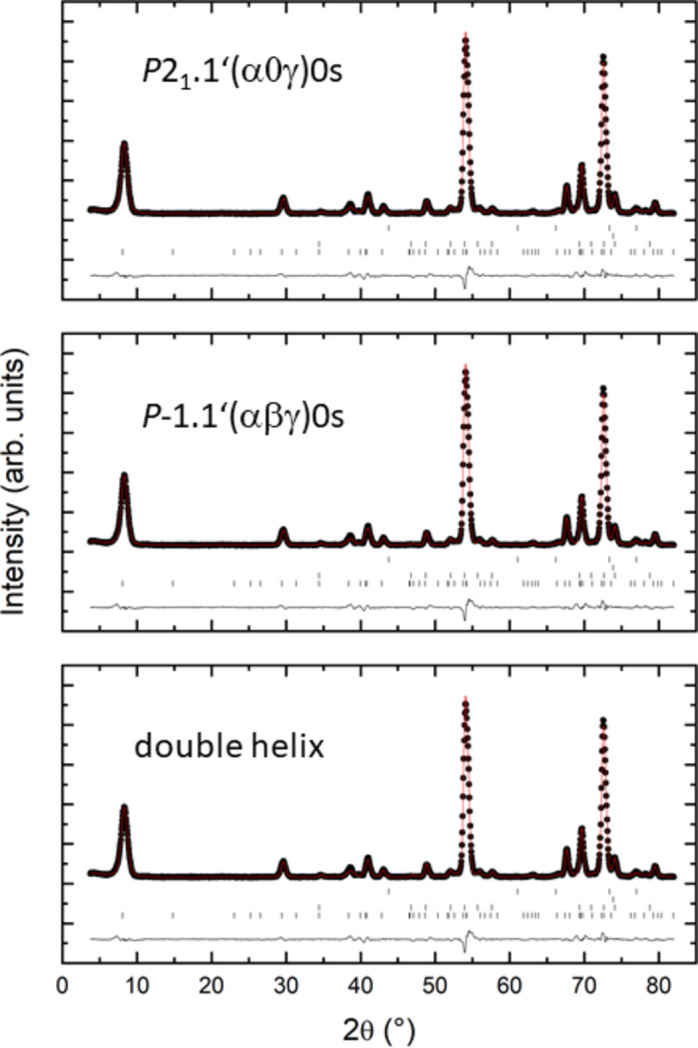
Measured, calculated and difference powder neutron pattern for selected models at 1.5 K (λ = 2.45 Å). Ticks indicate the positions of the reflections (from top to bottom) for Cr_2_O_3_, Cr, the high-temperature phase of CrAs and the low-temperature phase of CrAs, respectively.

The unrestrained magnetic models described above were refined on the basis of the neutron powder diffraction measurements (Table 3 ▸). All superspace groups pertaining to the nuclear *Pnma* symmetry and its subgroups were tested (Fig. 2 ▸). The results show that the models in *P*2_1_.1′(α0γ)0*s* and in *P* 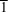 .1′(αβγ)0*s*, which also lead to very good agreement factors for the single-crystal data, show the best fit. Agreement factors for the double-helical model described in the literature are slightly worse. In addition, the differences in agreement factors with some other superspace groups are quite small.

Powder data measured at higher temperatures (240 K) correspond to the highest measured temperature in the magnetically ordered phase below the anti-isostructural phase transition. Only those superspace groups with satisfactory agreement factors both for the single-crystal data at 0.12 GPa and for powder data at the lowest temperature were considered in addition to the double-helix literature model. Here, the model in *P*2_1_.1′(α0γ)0*s* gives the best agreement factors. However, the differences between this and the other refined models are very small.

The models corresponding to *P*2_1_/*n*.1′(0βγ)0*ss* and *Pn*.1′(0βγ)*ss* exhibit consistently slightly worse agreement for the satellite reflections at both temperatures and can be discarded on the basis of the powder refinements. In contrast to the single-crystal data, the agreement of the double-helix model with the data is not significantly worse than for the other remaining superspace groups.

## 4. Discussion

The results presented here indicate that, while the double-helix model (Watanabe *et al.*, 1969 ▸; Selte *et al.*, 1971 ▸) indeed leads to a satisfactory fit of the powder diffraction data, the same is not true for the single-crystal data. Based on this observation, we conclude that this model is in fact incorrect. Although our single-crystal data were measured under pressure, there is no indication of an additional magnetic or structural phase transition within the antiferromagnetic phase region of CrAs up to about 0.84 GPa and 2 K (Shen *et al.*, 2016 ▸; Grzechnik *et al.*, 2023 ▸), so that we generalize our findings on the magnetic structure of CrAs to the whole corresponding stability region of the antiferromagnetically ordered phase.

**Figure 3:**
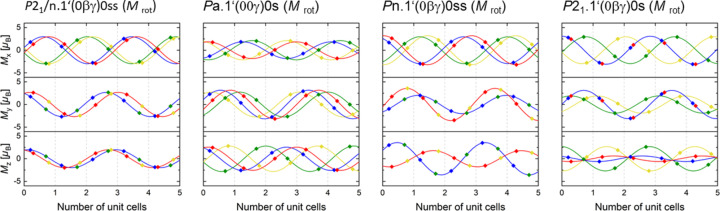
The directional components of the magnetic moment along the *x*, *y* and *z* axes (*M*_*x*_, *M*_*y*_ and *M*_*z*_, respectively) in the approximate structures with *c*′ = 20/7 *c* of the basic structures for the magnetic models in *P*2_1_/*n*.1′(0βγ)0*ss*, *Pa*.1′(00γ)0*s*, *Pn*.1′(0βγ)0*ss* and *P*2_1_.1′(0βγ)0*s* at 0.12 GPa and 2 K. For the sake of clarity, the components are plotted only for five unit cells. Points indicate values realized on Cr sites, full lines show the underlying modulation function. The colours correspond to different Cr sites. Blue, red, green and yellow symbols/lines are for CrI (*x*, *y*, *z*), CrII (*x* + ½, −*y* + ½, −*z* + ½), CrIII (−*x* + ½, −*y*, *z* + ½) and CrIV (−*x*, *y* + ½, −*z*), respectively. The dashed grey lines mark the unit-cell borders of the basic crystal structure. The absolute values |*M*| of the magnetic moment are constant: |*M*| = 3.17 (3) μ_B_ for *P*2_1_/*n*.1′(0βγ)0*ss*, |*M*| = 3.18 (2) μ_B_ for *Pa*.1′(00γ)0*s*, |*M*| = 3.59 (6) μ_B_ for *Pn*.1′(0βγ)0*ss* and |*M*| = 3.15 (2) μ_B_ for *P*2_1_.1′(0βγ)0*s*.

Table 4 ▸ shows the magnetic moment components at all Cr sites for the selected models restricted to a pure rotation of the magnetic moments. Figs. 3 ▸ and 4 ▸ show the modulation of the magnetic moments of Cr with a breakdown of the magnetic components along the *x*, *y* and *z* axes for selected models, assuming the condition that the absolute value |*M*| of the magnetic moments on all Cr atoms is constant. The modulation was constructed over 20 nuclear unit cells containing seven period lengths of the modulation in a good approximation of the incommensurate propagation vector **k** with *k*_c_ ≃ 0.35 = 7/20. Remarkably, the absolute values of the magnetic moment are about 3.2 μ_B_ for most of these models. Only in *Pn*.1′(0βγ)0*ss* and the double helix are they 3.59 (6) and 2.8859 (8) μ_B_, respectively. The values of 3.2–3.6 μ_B_ for the magnetic moment of the Cr atoms indicate that the previously assumed value of 1.7 μ_B_ (Zavadskii & Sibarova, 1980 ▸) is severely underestimated.

As can be seen from Table 1 ▸, agreement factors for the satellite reflections are best for the model *P*2_1_.1′(α0γ)0*s*, and a Hamilton test (Hamilton, 1965 ▸) carried out on the different models confirms its superiority over the alternatives. Since agreement factors for part of the other models are only slightly worse, we include them also as they might provide valuable input for future comparative theoretical investigations. It is noteworthy that, in contrast to the other candidate models, the agreement factors for the model *P*2_1_.1′(α0γ)0*s* (rot) do increase only slightly when one introduces the additional restrictions to the refinement (this way decreasing the number of magnetic parameters in the refinement).

A further observation, which would support this model as being the correct one, follows from the relationship between the modulation vector and the direction of the rotation of the spins. In CrAs, the irrational component of the modulation vector runs parallel to c*. If the ordering of the spins was a pure helix, the unit vector perpendicular to the spin rotation plane should be parallel to c* (which is the case in the double-helix model from the literature). If the ordering of the spins was cyclo­idal, the unit vector perpendicular to the spin rotation plane should be perpendicular to c*. However, in all of the refined models of the magnetic structure of CrAs, which lead to the best agreement factors, the orderings of the spins of CrAs cannot be described as a pure helix nor as a pure cycloid. Instead, the spin rotation planes make an angle α, 0° < α < 90°, with respect to the c* direction. This can be clearly seen from Table 3 ▸, which shows the magnetic moment components at all Cr sites for the discussed models. The conditions for the superspace groups allowing for a helical or cyclo­idal ordering, in which all symmetry-related magnetic moments in the lattice rotate in the same direction, *i.e.* with the same chirality, have been presented by Fabrykiewicz *et al.* (2021 ▸). While helical ordering is compatible with the superspace groups derived from the crystal classes 1, 2, 222, 4, 422, 3, 32, 6 and 622, the cyclo­idal ordering is allowed in the magnetic superspace groups derived from crystal classes 1, 2, *m* and *mm*2. Of all the crystal classes only two, 1 and 2, allow for both helical and cycloid orderings. It is striking that crystal class 2 is exactly the one that corresponds to the model in magnetic superspace group *P*2_1_.1′(α0γ)0*s*.

**Figure 4:**
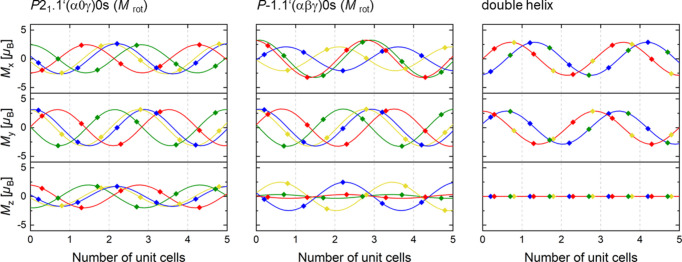
The directional components of the magnetic moment along the *x*, *y* and *z* axes (*M*_*x*_, *M*_*y*_ and *M*_*z*_, respectively) in the approximate structures with *c*′ = 20/7 *c* of the basic structures for the magnetic models in *P*2_1_.1′(α0γ)0*s*, *P* 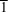 .1′(αβγ)0*s* and in the double helix (Watanabe *et al.*, 1969 ▸; Selte *et al.*, 1971 ▸) at 0.12 GPa and 2 K. For the sake of clarity, the components are plotted only for five unit cells. Points indicate values realized on Cr sites, full lines show the underlying modulation function. The colours correspond to different Cr sites. Blue, red, green and yellow symbols/lines are for CrI (*x*, *y*, *z*), CrII (*x* + ½, −*y* + ½, −*z* + ½), CrIII (−*x* + ½, −*y*, *z* + ½) and CrIV (−*x*, *y* + ½, −*z*), respectively. The dashed grey lines mark the unit-cell borders of the basic crystal structure. The absolute values |*M*| of the magnetic moment are constant: |*M*| = 3.16 (3) μ_B_ for *P*2_1_.1′(α0γ)0*s*, |*M*| = 3.23 (4) μ_B_ for *P* 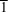 .1′(αβγ)0*s* and |*M*| = 2.8859 (8) μ_B_ for the double helix.

A notable difference between the magnetic models in Fig. 3 ▸ and the double-helix literature model (Fig. 4 ▸) is the vanishing *M*_*z*_ component in the double-helix model forced by the assumption that the magnetic structure of CrAs is coplanar. The confinement of the magnetic moment of the Cr atoms to the (*a*, *b*) plane postulated by Watanabe *et al.* (1969 ▸) and Selte *et al.* (1971 ▸) for the double-helix model does not explain the intensity distribution of the satellite reflections measured on a single crystal. In addition, even if considering only the projection of the models along the *c* axis, the double-helix model is not replicated by the other models. For the presence of two in-phase helices as described in the double-helix model, the conditions are: (i) to realize a circular helix, *M*_*x*_ and *M*_*y*_ must have the same amplitude and a phase difference of 90°, and (ii) to realize in-phase helices on different Cr sites, the relevant modulations have to be in-phase. Regarding the latter condition, the double-helix model is fundamentally incompatible with all but the *P*1.1′(αβγ)0*s* model.

## 5. Conclusions

Models of the incommensurate magnetic structure of CrAs are derived using group-theoretical considerations and refined using the concept of magnetic superspace groups. In the literature, the underlying magnetic structure is described as a double helix propagating along the *c* axis (Watanabe *et al.*, 1969 ▸; Selte *et al.*, 1971 ▸). On the basis of the neutron single-crystal data, it is concluded that the double-helix model from the literature is erroneous as it does not reproduce the intensities of the satellite reflections. Instead, several new models for the magnetic structure in CrAs are derived. Each of them is spiral-like (rotating constant magnetic moment), with no spin-density wave character that would cause a variable magnetic moment. The magnetic moments have directional components in all three directions. The ordering of the spins is neither a pure helix, where the unit vector perpendicular to the spin rotation plane is parallel to c*, nor a pure cycloid, where the unit vector perpendicular to the spin rotation plane is perpendicular to c*. Instead, in all of the models the unit vectors of the spin rotation planes make an angle α, 0° < α < 90°, with respect to the c* direction. From the candidate models, the one in superspace group *P*2_1_.1′(α0γ)0*s* yields the best agreement factors in the refinements of the neutron single-crystal and powder diffraction data. It is the only one in which all the magnetic moments rotate with the same chirality.

Our results provide a basis for future investigations of the magnetic interactions and spin excitations in CrAs using experimental methods (like inelastic neutron scattering and polarized neutron scattering) complemented with theoretical calculations, both being beyond the scope of the present work.

## 6. Related literature

The following references are cited in the supporting information: Ivantchev *et al*. (2000 ▸), Wilkinson *et al*. (1988 ▸).

## Supplementary Material

Crystal structure: contains datablock(s) global, I, I_1, I_2, I_3, I_4, I_5, I_6, I_7. DOI: 10.1107/S205252062300817X/xk5103sup1.cif

Structure factors: contains datablock(s) global, I. DOI: 10.1107/S205252062300817X/xk5103Isup2.hkl

Supporting information. DOI: 10.1107/S205252062300817X/xk5103sup3.pdf

ZTRsboatsIh

CCDC references: 2295689, 2300156, 2300157, 2300158, 2300159, 2300160, 2300161, 2300162

## Figures and Tables

**Table 1:** Final agreement factors for the best tested magnetic models for CrAs based on the refinement of the neutron single-crystal data at 0.12 GPa and 2 K The superscripts [100], [010] and [001] indicate the direction in which the twofold screw axis of the respective space group *P*2_1_ is oriented in order to allow differentiation. A space-group symbol in parentheses indicates a subgroup of *Pnma*.

				All		Satellites		|*M*|		
Nuclear space group	Magnetic superspace group	Restraints	No. magnetic parameters	*R* (obs/all) (%)	*wR* (obs/all) (%)	*R* (obs/all) (%)	*wR* (obs/all) (%)	Min.	Max.	<|*M*|_max_ (Cr^3+^)
(*P*2_1_/*n*)	*P*2_1_/*n*.1′(0βγ)0*ss*	–	6	8.13/12.51	9.33/9.96	21.85/38.38	28.62/29.86	2.6 (2)	3.6 (3)	Yes
		*M*_rot_	5	8.45/12.84	10.00/10.63	28.92/45.54	32.93/34.17	3.17 (9)		Yes
(*Pn*)	*Pn*.1′(0βγ)0*ss*	–	12	8.64/14.77	8.79/9.72	22.88/60.38	19.44/22.41	Refinement unstable		
		*M*_equal_	10	8.73/14.81	9.22/10.06	25.35/61.37	22.71/24.86	1.6 (3)	5.0 (3)	No
		*M*_rot_	10	8.83/14.63	10.35/11.03	28.70/57.76	29.96/31.10	3.59 (9)		Yes
(*P*2_1_^[100]^)	*P*2_1_.1′(0βγ)0*s*	–	12	8.29/13.97	8.47/9.27	19.04/49.01	19.97/21.83	0.20 (16)	6.00 (16)	No
		*M*_equal_	10	8.34/14.03	8.68/9.45	20.57/50.92	21.58/23.23	1.7 (2)	4.2 (2)	No
		*M*_rot_	9	8.55/14.30	9.14/9.88	28.42/59.22	25.00/26.40	3.17 (6)		Yes
(*P*2_1_^[010]^)	*P*2_1_.1′(α0γ)0*s*	–	12	8.95/15.72	8.35/9.17	17.18/45.36	14.58/16.86	1.2 (14)	4.4 (14)	No
		*M*_equal_	10	8.97/15.82	8.42/9.30	17.66/47.56	15.48/18.25	2.29 (11)	3.90 (11)	Yes
		*M*_rot_	9	9.06/15.92	8.65/9.47	20.22/50.05	17.86/19.97	3.16 (8)		Yes
(*Pa*)	*Pa*.1′(00γ)0*s*	–	12	8.13/14.10	8.37/8.94	21.62/52.38	19.57/21.23	2 (2)	5.0 (9)	No
		*M*_equal_	10	8.17/14.11	8.49/9.03	22.61/52.77	20.53/21.94	1.4 (2)	4.3 (3)	No
		*M*_rot_	9	8.31/14.28	8.82/9.35	27.15/57.37	22.95/24.24	3.18 (6)		Yes
(*P* 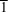 )	*P* 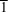 .1′(αβγ)0*s*	–	12	8.74/15.49	8.48/9.47	21.31/51.17	17.43/20.33	1.3 (6)	4.6 (6)	No
		*M*_equal_	10	8.94/15.71	8.94/9.84	27.31/56.48	21.72/23.71	2.46 (16)	3.89 (14)	Yes within error
		*M*_rot_	9	8.98/15.90	9.11/10.01	27.92/60.49	23.11/25.10	3.23 (9)		Yes
(*P*1)	*P*1.1′(αβγ)0*s*	–	24	8.75/16.02	8.80/9.93	20.69/61.06	18.26/21.37	1 (6)	7 (5)	No
		*M*_equal_	18	8.71/16.09	8.93/10.22	19.36/62.84	19.36/23.59	2.3 (2)	4.7 (1)	No
		*M*_rot_	17	8.87/16.24	9.32/10.49	23.82/66.16	22.34/25.58	3.70 (6)		Yes
	Double helix ↑*c*	Helix	2	10.28/17.22	14.39/15.41	64.65/89.49	51.33/53.63	2.89 (1)		Yes

**Table 2:** Representative operations of the three magnetic superspace groups leading to the best refinement results The generalized Seitz-type symbols (left column) and the symmetry codes as used in the program *Jana2006* are given.

*P*2_1_/*n*.1′(0βγ)0*s*					
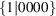	*x*1	*x*2	*x*3	*x*4	*m*
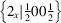	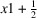	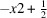	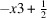	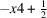	*m*
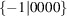	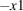	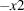	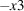	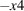	*m*
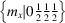	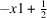	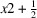	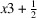	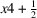	*m*
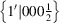	*x*1	*x*2	*x*3	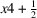	−*m*
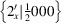	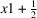	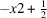	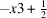	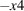	−*m*
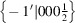	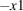	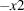	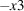	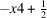	−*m*
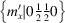	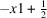	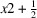	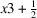	*x*4	−*m*
					
*Pn*.1′(0βγ)0*s*					
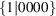	*x*1	*x*2	*x*3	*x*4	*m*
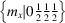	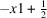	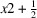	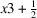	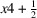	*m*
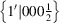	*x*1	*x*2	*x*3	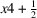	−*m*
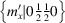	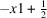	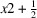	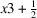	*x*4	−*m*

*P*2_1_.1′(0βγ)0*s*					
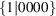	*x*1	*x*2	*x*3	*x*4	*m*
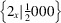	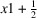	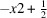	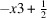	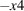	*m*
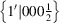	*x*1	*x*2	*x*3	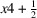	−*m*
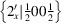	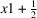	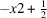	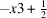	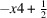	−*m*

*P*2_1_.1′(α0γ)0*s*					
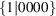	*x*1	*x*2	*x*3	*x*4	*m*
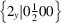	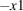	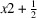	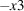	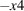	*m*
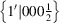	*x*1	*x*2	*x*3	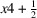	−*m*
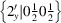	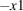	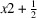	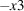	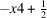	−*m*

*Pa*.1′(00γ)0*s*					
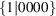	*x*1	*x*2	*x*3	*x*4	*m*
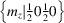	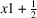	*x*2	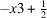	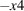	*m*
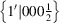	*x*1	*x*2	*x*3	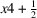	−*m*
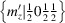	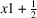	*x*2	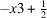	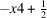	−*m*

*P* 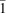 .1′(αβγ)0*s*					
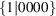	*x*1	*x*2	*x*3	*x*4	*m*
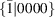	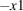	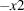	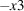	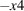	*m*
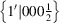	*x*1	*x*2	*x*3	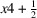	−*m*
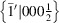	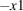	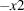	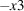	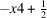	−*m*

**Table 3:** Final agreement factors for selected magnetic models based on the refinement of neutron powder data at two representative temperature points

			Satellites		Profile	
*T* (K)	Nuclear space group	Magnetic superspace group	*R* (obs/all) (%)	*wR* (obs/all) (%)	*R*_p_ (%)	*wR*_p_ (%)
1.5	*P*2_1_/*n*	*P*2_1_/*n*.1′(0βγ)0*ss*	7.22/7.94	6.48/6.51	6.33	8.61
	*Pn*	*Pn*.1′(0βγ)*ss*	7.04/7.49	6.64/6.68	6.29	8.49
	*P*2_1_^[100]^	*P*2_1_.1′(0βγ)0*s*	5.11/5.21	5.37/5.38	5.87	7.94
	*P*2_1_^[010]^	*P*2_1_.1′(α0γ)0*s*	4.97/5.07	5.29/5.29	5.85	7.90
	*Pa*	*Pa*.1′(00γ)0*s*	5.83/5.83	5.49/5.51	5.97	8.09
	*P* 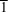	*P* 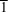 .1′(αβγ)0*s*	4.70/4.78	4.81/4.82	5.71	7.80
	*P*1	*P*1.1′(αβγ)0*s*	4.94/5.22	5.24/5.27	5.78	7.94
		Double helix ↑*c*	4.97/5.80	5.13/5.20	5.99	8.22
240	*P*2_1_/*n*	*P*2_1_/*n*.1′(0βγ)0*ss*	6.35/6.90	5.78/5.79	6.95	9.14
	*Pn*	*Pn*.1′(0βγ)*ss*	7.04/7.62	6.18/6.20	7.10	9.27
	*P*2_1_^[100]^	*P*2_1_.1′(0βγ)0*s*	5.05/5.25	5.11/5.12	6.94	9.04
	*P*2_1_^[010]^	*P*2_1_.1′(α0γ)0*s*	4.95/5.49	4.98/5.01	6.78	8.83
	*Pa*	*Pa*.1′(00γ)0*s*	5.56/5.82	5.16/5.17	6.91	9.00
	*P* 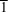	*P* 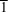 .1′(αβγ)0*s*	5.46/5.87	5.08/5.09	6.70	8.74
	*P*1	*P*1.1′(αβγ)0*s*	5.16/5.85	5.13/5.17	6.78	8.86
		Double helix ↑*c*	5.23/5.83	5.03/5.08	6.93	9.00

**Table 4:** Magnetic moment components in the models allowing only for a rotation of the magnetic moments of the incommensurately modulated structure of CrAs

	*x*sin1	*x*cos1	*y*sin1	*y*cos1	*z*sin1	*z*cos1
*P*2_1_/*n*.1′(0βγ)0*s*						
Cr1	2.93 (2)	0.59 (4)	−1.10 (4)	2.45 (3)	0.51 (6)	1.92 (4)
						
*Pn*.1′(0βγ)0*s*						
Cr1_1	−1.45 (6)	2.59 (4)	1.18 (7)	−1.65 (6)	3.07 (2)	1.86 (4)
Cr1_2	−0.17 (6)	−3.21 (3)	3.53 (2)	−0.52 (6)	−0.81 (9)	−1.60 (7)
						
*P*2_1_.1′(0βγ)0*s*						
Cr1_1	0.02 (4)	3.106 (19)	3.130 (15)	0.04 (4)	−0.34 (14)	0.51 (11)
Cr1_2	−2.938 (18)	−0.46 (4)	1.12 (5)	−1.55 (4)	−0.14 (4)	−2.70 (2)
						
*P*2_1_.1′(α0γ)0*s*						
Cr1_1	−2.46 (2)	0.39 (5)	0.45 (4)	3.057 (5)	−1.81 (3)	0.23 (6)
Cr1_2	−0.05 (4)	2.64 (2)	−3.075 (7)	0.12 (4)	−0.31 (6)	−1.61 (4)
						
*Pa*.1′(00γ)0*s*						
Cr1_1	0.72 (6)	−1.68 (4)	2.762 (13)	1.07 (3)	−0.80 (5)	2.20 (3)
Cr1_2	−0.83 (4)	−2.13 (3)	1.53 (3)	−1.99 (3)	−2.40 (3)	−0.53 (4)
						
*P* 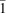 .1′(αβγ)0*s*						
Cr1_1	1.95 (3)	−0.02 (6)	0.37 (4)	3.020 (7)	−2.32 (3)	0.47 (5)
Cr1_2	−0.51	3.000 (15)	3.000 (15)	0.53 (3)	−0.28 (17)	0.24 (16)
