# Supplementary material for: Incommensurate magnetic structure of CrAs at low temperatures and high pressures
Source: Acta Crystallogr B Struct Sci Cryst Eng Mater. 2023 Oct 12;79(Pt 6):473–81. doi: 10.1107/S205252062300817X (PMC10833358; doi:10.1107/S205252062300817X)
Supplement: Supplementary file 3 [file b-79-00473-sup3.pdf]

STRUCTURAL SCIENCE
CRYSTAL ENGINEERING
MATERIALS

Volume 79 (2023)

Supporting information for article:

Incommensurate magnetic structure of CrAs at low temperatures and high pressures

Andreas Eich, Andrzej Grzechnik, Yixi Su, Bachir Ouladdiaf, Denis Sheptyakov, Thomas Wolf, Vaclav Petricek, Hend Shahed and Karen Friese

S1. Analysis of the neutron powder diffraction data

The neutron powder data obtained from the experiments at HRPT (Figure S1) were refined using Jana2020 (Petricek *et al*., 2023) in three steps. In the first step, a Le Bail fit of the powder pattern was made, in which the positions of the magnetic reflections were described only by an incommensurate propagation vector $\boldsymbol{k}$ of the form (0, 0, k_c). The lattice parameters a, b, and c, the k_c component of the propagation vector $\boldsymbol{k}$, the peak-shape parameters GW and LY, the shift parameter shift and the strain tensor parameter St0022 were refined. The background was refined with about 30 manually set points and 5 refined Legendre polynomial terms. As the data showed elemental chromium and Cr_2O_3 to be present, this was taken into account as additional phases. In the second step, the nuclear structure was determined by the Rietveld method, for which the magnetic reflections were masked when possible. The refinement was carried out in space group *Pnma* in accordance with our single crystal data. The powder data showed additionally the presence of CrAs in the paramagnetic high-temperature phase, which was taken into account by refining its lattice parameters and a corresponding phase volume fraction. In the third step, the refined nuclear structure, in combination with the deduced propagation vector, was taken as basis for the subsequent derivation of the magnetic superspace groups using the built-in algorithms from Jana2020 (Petricek *et al*., 2023). Refinements of the magnetic structure were carried out in all derived superspace groups. As the fit of the magnetic reflections using the magnetic superspace models derived from the *Pnma* of the nuclear structure did not lead to a satisfactory agreement, it was necessary to test also lower symmetries. For this, the space group in which the nuclear refinement was carried out was reduced to lower-symmetrical space groups. However, in all the lower-symmetrical descriptions the higher *Pnma* symmetry was retained by fixing the respective atomic coordinates using local symmetry operations. For the refinements of the magnetic structures in all considered magnetic superspace groups, all parameters corresponding to the nuclear structure were fixed and not refined. The refined

parameters were in all cases the magnetic modulation amplitudes and 5 background parameters. For those structures, where multiple Cr positions are present, the magnetic modulation amplitudes were in general not restricted, the only exception being the model corresponding to the double-helical structure reported in the literature (Watanabe *et al*., 1969).

S2. Analysis of the neutron single-crystal diffraction data

The ω-scans consisted of 31 single frames centred around the set value for ω. The indexing of the reflections was based on the refined orientation matrix. The reflections were then integrated using the method described by Wilkinson *et al*. (1988). The magnetic reflections were measured on the basis of a magnetic propagation vector of $\boldsymbol{k}$ = (0, 0, 0.353(2)). The measured magnetic reflections were integrated in the same way as the nuclear ones. In a first step, the list of integrated intensities of the nuclear reflections was subjected to the intensity correction for the transmission loss due to the used clamp cell (Figure S2). The corrected intensities were then used as a basis for the refinement of the nuclear structure with Jana2020 (Petricek *et al*., 2023). The refinements of the nuclear structure at both pressure points were carried out in space group *Pnma*. Refined parameters were the free atom coordinates, the anisotropic displacement parameters, an isotropic extinction coefficient G_{iso}, and the twin volume fractions *twvol2* and *twvol3*.

The intensity of the magnetic reflections was corrected for the transmission losses in the same way as the nuclear reflections. On the basis of the refined nuclear structure at both pressure points and the propagation vectors, the magnetic models corresponding to different magnetic superspace groups were derived using the built-in algorithms of Jana2020 (Petricek *et al*., 2023). To determine the magnetic structure, the data were refined in all magnetic superspace groups allowed by the nuclear space group *Pnma*, as well as all the *translationengleiche* subgroups of *Pnma* down to *P*1. For the refinement of the magnetic structure, the nuclear

structure was fixed to the values obtained from the nuclear refinement, and the symmetry restrictions imposed by *Pnma* were applied also in the cases with lower symmetry. In all the tested magnetic superspace groups, the atoms were treated anisotropically. The twin volume fractions (*twvol2* and *twvol3*) and the magnetic modulation amplitudes not restricted by the symmetry were refined. In cases, where the lower symmetry leads to a splitting of the atom positions, the harmonic displacement parameters were restricted to follow the higher symmetry with the help of local symmetry operations. For those structures where multiple Cr positions are present, the magnetic modulation amplitudes were in general not restricted, the only exception being the model which corresponds to the double-helical structure reported in the literature (Watanabe *et al*., 1969).

References

Petříček, V., Palatinus, L., Plášil, J. & Dušek, M. (2023), Jana2020 – a new version of the crystallographic computing system Jana, Z. Kristallogr. – Cryst. Mat., in print, 10.1515/zkri-2023-0005.

Watanabe, H., Kazama, N., Yamaguchi, Y., Ohashi, M. (1969), Magnetic structure of CrAs and Mn-substituted CrAs, *J. Appl. Phys.* **40** (1969) 1128–1129, 10.1063/1.1657559

Wilkinson, C., Khamis, H.W., Stansfield, R.F.D., McIntyre, G.J. (1988), Integration od single-crystal reflection using area multidetectors, Acta Cryst. 21, 471-478.

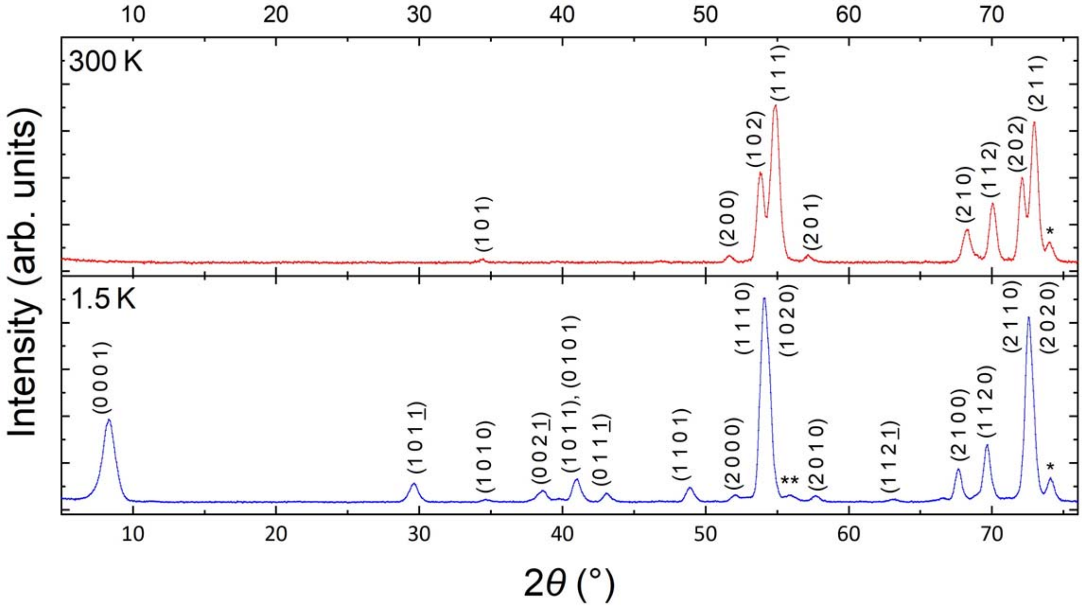

Figure S1 Neutron powder diffraction patterns measured at 300 K (*top*) and at 1.5 K (*bottom*). At 1.5 K, the reflections are indexed in (3+1)-dimensional superspace with the propagation vector ***k*** = (0, 0, γ). * marks a reflection belonging to the Cr impurity. ** marks a reflection belonging to the still-present high-temperature phase.

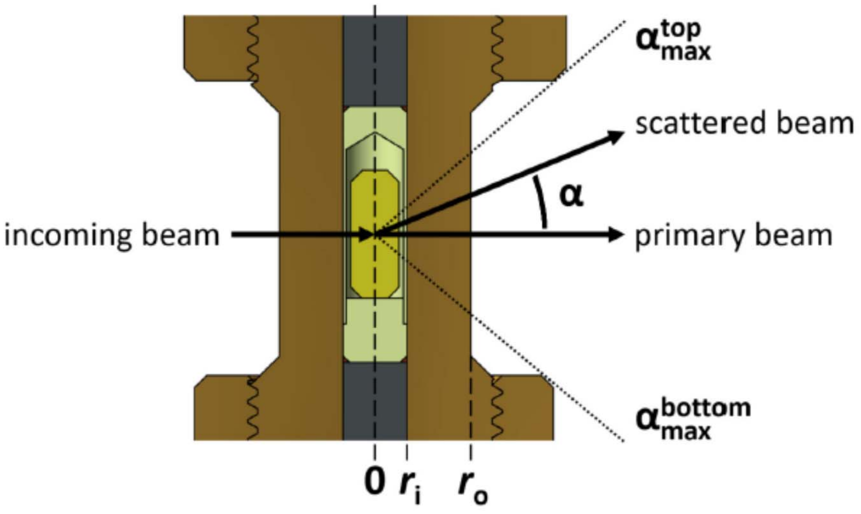

Figure S2 The geometrical model for the absorption correction of the data measured in the clamp cell.

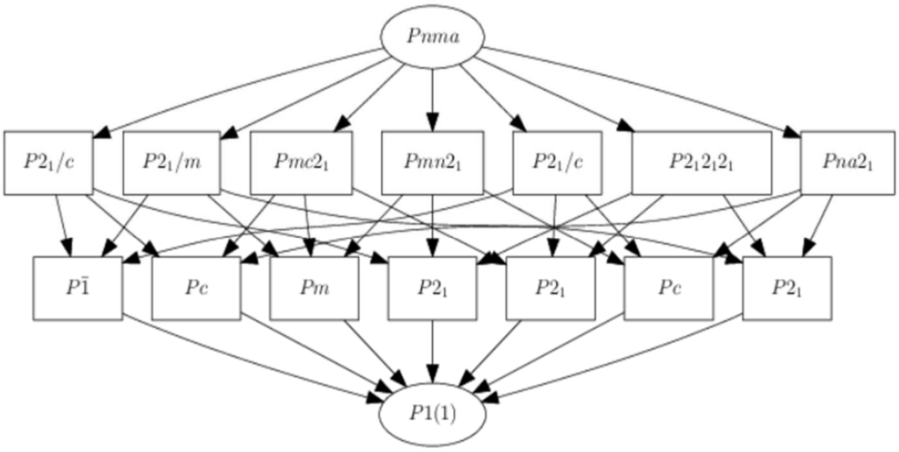

Figure S3 *Translationengleiche s*ubgroups of Pnma used for the derivation of the magnetic superspace groups for the refinement of the magnetic structure of CrAs (drawn with the program Subgroupgraph (Ivantchev et. al., 2000, *J. Appl. Cryst.* **33**, 1190-1191**).**

Table S1 Final agreement factors for the neutron single-crystal data of CrAs at 0.12 GPa and 2 K for the magnetic models derived from nuclear space group *Pnma*.

		all		main		satellites	
Nuclear space group	Magnetic superspace group	R (obs/all)	wR (obs/all)	R (obs/all)	wR (obs/all)	R (obs/all)	wR (obs/all)
Pnma	$Pnma.1'$(00g)000s	9.95/13.12	22.55/22.66	7.23/10.04	6.21/6.50	80.45/89.37	92.51/92.62
	$Pnma.1'$(00g)ss0s	9.00/12.51	16.94/17.16	7.23/10.04	6.21/6.50	54.88/73.71	67.39/67.90
	$Pnma.1'$(00g)0s0s	8.37/11.55	10.08/10.31	7.23/10.04	6.21/6.50	37.96/48.99	34.37/34.65
	$Pnma.1'$(00g)s00s	9.66/12.93	15.01/15.23	7.23/10.04	6.21/6.50	72.69/84.56	58.53/58.96

Table S2 Final agreement factors for the neutron single-crystal data of CrAs at 0.12 GPa and 2 K for all tested magnetic superspace groups derived from the *t*-subgroups of *Pnma.* The superscripts [100], [010] and [001] do not belong to the formal space group symbol, but indicate the direction in which the two-fold screw axis of the respective space group $P2_1$ is oriented in order to allow differentiation. A space group symbol in parentheses indicates a subgroup of *Pnma*.

$(P2_1ma)$	$P2_1ma.1'(00g)000s$	8.30/13.27	10.35/10.76	7.41/11.26	6.53/6.90	33.48/63.54	33.57/34.51
	$P2_1ma.1'(00g)0s0s$	8.16/13.15	10.03/10.47	7.41/11.26	6.53/6.90	29.18/60.51	31.90/32.97
$(Pn2_1a)$	$Pn2_1a.1'(00g)000s$	8.25/12.66	10.58/10.94	7.15/11.01	6.47/6.97	34.38/49.72	38.02/38.34
	$Pn2_1a.1'(00g)s00s$	8.12/12.60	9.48/9.85	7.14/11.01	6.47/6.97	31.22/48.38	31.66/31.86
$(Pnm2_1)$	$Pnm2_1.1'(00g)000s$	10.32/15.26	24.05/24.28	7.77/11.95	7.18/7.60	78.42/93.01	93.01/93.07
	$Pnm2_1.1'(00g)ss0s$	9.03/14.11	17.19/17.44	7.77/11.95	7.18/7.60	42.70/65.53	63.49/63.62
	$Pnm2_1.1'(00g)0sss$	9.08/14.29	11.92/12.44	7.77/11.95	7.18/7.60	44.02/70.16	39.11/40.35
	$Pnm2_1.1'(00g)s0ss$	8.79/14.27	10.77/11.21	7.77/11.95	7.18/7.60	35.96/69.60	33.24/33.98
$(P2_12_12_1)$	$P2_12_12_1.1'(00g)000s$	8.67/13.52	14.05/14.39	7.86/12.22	6.82/7.31	32.39/48.64	55.48/55.77
	$P2_12_12_1.1'(00g)00ss$	8.69/13.68	9.66/10.06	7.86/12.22	6.82/7.31	33.02/53.06	31.39/31.67
$(P2_1/n)$	$P2_1/n.1'(0bg)00s$	8.66/13.30	13.24/13.76	7.38/11.32	6.68/7.31	48.16/68.77	52.70/53.56
	$P2_1/n.1'(0bg)0ss$	7.83/12.25	9.01/9.66	7.38/11.32	6.68/7.31	21.64/38.26	28.43/29.69
$(P2_1/m)$	$P2_1/m.1'(a0g)00s$	8.75/14.75	9.57/10.14	7.93/13.02	7.16/7.71	30.64/55.88	28.66/29.64
	$P2_1/m.1'(a0g)0ss$	8.90/14.66	11.33/11.76	7.93/13.02	7.16/7.71	35.01/53.53	39.08/39.33
$(P2_1/a)$	$P2_1/a.1'(00g)00s$	9.06/13.63	16.33/16.65	7.37/11.19	6.55/6.88	50.45/70.36	66.25/67.07
	$P2_1/a.1'(00g)s0s$	8.30/12.56	8.61/8.94	7.37/11.19	6.55/6.88	31.02/44.43	25.51/26.02
(Pn)	$Pn.1'(0bg)0s$	8.76/14.96	10.22/11.00	7.84/12.43	7.32/7.98	32.50/68.85	30.70/32.35
	$Pn.1'(0bg)ss$	8.39/14.47	8.50/9.44	7.84/12.43	7.32/7.98	22.52/58.07	19.50/22.34
$(P2_1{}^{[100]})$	$P2_1.1'(0bg)0s$	8.02/13.73	8.12/8.93	7.70/12.54	6.92/7.62	18.84/48.77	19.85/21.69
(Pm)	$Pm.1'(a0g)0s$	8.49/14.99	9.03/9.83	7.84/12.88	7.41/8.03	26.24/65.29	22.33/24.34
	$Pm.1'(a0g)ss$	8.77/15.17	10.99/11.63	7.84/12.88	7.41/8.03	34.04/69.84	33.93/35.09
$(P2_1{}^{[010]})$	$P2_1.1'(a0g)0s$	8.70/15.50	8.02/8.86	8.38/14.12	7.55/8.24	17.09/45.50	14.44/16.77
(Pa)	$Pa.1'(00g)0s$	7.77/13.86	7.80/8.43	7.28/12.27	6.52/7.00	21.29/52.56	19.53/21.25
$(P2_1{}^{[001]})$	$P2_1.1'(00g)0s$	9.04/16.78	16.78/17.20	7.95/13.00	7.39/8.02	37.72/62.45	62.78/62.97
	$P2_1.1'(00g)ss$	8.67/15.27	8.95/9.83	7.95/13.00	7.39/8.02	27.66/64.57	22.17/24.66
$(P\bar{1})$	$P\bar{1}.1'(abg)0s$	8.46/15.25	8.14/9.15	8.01/13.71	7.39/8.18	21.12/51.12	17.28/20.21
$(P1)$	$P1.1'(abg)0s$	8.51/15.78	8.53/9.67	8.07/13.77	7.54/8.44	20.69/60.85	18.43/21.46
	double helix $\uparrow c$	10.05/17.02	14.16/15.20	8.07/13.77	7.54/8.44	64.61/89.86	51.08/53.45
